# Impact of Left Ventricular‐Vascular Interaction on Long‐Term Outcome After Heart Transplantation

**DOI:** 10.1111/ctr.70178

**Published:** 2025-05-13

**Authors:** Mattia Corianò, Nicola Pradegan, Andrea Golfetto, Vincenzo Tarzia, Annalisa Angelini, Antonio Gambino, Chiara Tessari, Marny Fedrigo, Giuseppe Toscano, Gino Gerosa, Francesco Tona

**Affiliations:** ^1^ Department of Cardiac Thoracic, Vascular Sciences and Public Health, University Hospital Padua Padua Italy; ^2^ Cardiac Surgery Unit, Department of Cardiac Thoracic, Vascular Sciences, and Public Health, University of Padua Padua Italy; ^3^ Pathology and Pathological Anatomy Unit, Department of Cardiac Thoracic, Vascular Sciences and Public Health, University of Padova Padua Padova Italy

**Keywords:** arterial elastance, heart transplant, pressure‐volume loop, ventricular‐arterial coupling, ventricular elastance

## Abstract

**Background and aim:**

To compare pressure‐volume (PV) derivative variables between HT patients and healthy controls and to assess their impact on long‐term outcome.

**Methods:**

In this single‐center retrospective study, HT patients surviving their first post‐HT year with left ventricular ejection fraction (LVEF) ≥50%, absence of allograft vasculopathy, and rejection were enrolled. PV variable surrogates were measured by transthoracic echocardiography and compared with healthy controls. The endpoint was cardiovascular mortality.

**Results:**

From 1985 to 2015, 345 patients were enrolled. Arterial elastance (Ea) and left ventricular end‐systolic elastance (Ees) were higher in HT recipients than in healthy controls (4.03 vs. 1.65, *p* < 0.0001 and 6.75 vs. 2.47, *p* < 0.0001, respectively), while ventricular arterial coupling (VAC) was similar between the two groups (0.66 vs. 0.59, *p* = 0.105). After a median of 11.3‐year follow‐up, 59 (17%) HT recipients died. VAC was not significantly associated with cardiac mortality (*p* = 0.074). Survival was lower in HT recipients with Ea > 4 mmHg/mL/m^2^ and Ees ≤ 6.75 mmHg/mL/m^2^, and both were independently associated with mortality risk after adjustment (Ea > 4 mmHg/mL/m^2^: HR 2.25 [95% CI 1.38–3.66], *p* = 0.013; Ees ≤ 6.75 mmHg/mL/m^2^: HR 3.70 [95% CI 1.95–7.06], *p* = 0.001).

**Conclusions:**

In HT recipients surviving the first year after transplantation with normal LVEF, high Ea, and low Ees values were independently associated with poorer outcomes in long‐term follow‐up.

**Abbreviations**
ACR: acute cellular rejection
CAV: cardiac allograft vasculopathy
DT: deceleration time
Ea: arterial elastance
EDV: end‐diastolic volume
Eed: end‐diastolic elastance
Ees: end‐systolic elastance
ESP: end‐systolic pressure
ESV: end‐systolic volume
HF: heart failure
HHD: hypertensive heart disease
HR: hazard ratio
HT: heart transplant
IQR: interquartile range
ISHLT: International Society for Heart and Lung Transplantation
LV: left ventricular
LVEF: left ventricular ejection fraction
LV_we_
: left ventricular work efficiency
PE: potential energy
PV: pressure‐volume
PVA: pressure‐volume area
SV: stroke volume
SW: stroke work
TDI: tissue doppler imaging
VAC: ventricular‐arterial coupling

## 1 Introduction

Despite an increase in the efficacy, quantity, and availability of heart failure (HF) therapies, survival after a diagnosis of HF has shown only modest improvement in the 21^st^ century [1]. This means that many patients with HF still progress to advanced HF [2]. For these patients, heart transplantation (HT) remains the gold standard therapy to improve quality of life and prognosis [3]. According to current reports, the median survival after HT accounts of 12.5 years, with graft failure representing a substantial risk factor for adverse outcome, showing a reported incidence of 29% after 10 years post‐HT [4, 5]. Graft dysfunction in HT has various etiologies, ranging from cardiac conditions (e.g., ischemic insult, rejection, allograft vasculopathy, diastolic dysfunction) to vascular conditions (systemic arterial hypertension, central aortic stiffness). Early detection of cardiac graft failure is challenging because left ventricular ejection fraction (LVEF) often is normal in asymptomatic patients [5, 6]. In this context, ventricular‐vascular interaction might play a central role in affecting outcome after HT, and its evaluation may aid in the early diagnosis of graft failure. This hypothesis was tested in a few studies, which demonstrated an increase in ventricular‐arterial coupling (VAC) and its components (left ventricular elastance [Ees], arterial elastance [Ea]) after HT, both at rest and under inotropic stress, mainly due to a disproportionate increase of Ea [7, 8, 9, 10]. However, an association between ventricular‐vascular physiology and cardiac energetic efficiency with long‐term prognosis after HT has not yet been evaluated. In this study, we aimed to compare pressure‐volume (PV) relationship derivate variables between patients after HT and healthy controls. We further examined whether disparities in VAC and its components are associated with cardiac mortality after HT.

## 2 Methods

### 2.1 Study Population and Clinical Variables

In this single‐center, retrospective, observational cohort study, we collected data from patients who underwent HT at the University Hospital of Padua between November 1985 and December 2015. Since the start of the HT program at our institution, all surviving HT patients are routinely admitted to the hospital 1 year after transplantation for standard follow‐up procedures, including echocardiography, coronary angiography, and endomyocardial biopsy (EMB). Data from this 1‐year follow‐up of admissions have been systematically stored since the beginning of the HT program.

Inclusion criteria were as follows: LVEF ≥ 50%; International Society for Heart and Lung Transplantation (ISHLT) Cardiac Allograft Vasculopathy (CAV) of grade 0 (no detectable angiographic lesion) or 1 (angiographic left main <50%, or primary vessel with maximum lesion of 70% or any branch stenosis <70% without allograft dysfunction) at 1 year post‐HT [11]; ISHLT Acute Cellular Rejection (ACR) of grade 0 (no rejection) or 1R (mild rejection, interstitial, and/or perivascular infiltrate with up to one focus of myocyte damage) at 1 year post‐HT [12]. Patients who died within 1 year after HT were excluded. For further details, see Supporting Appendix.

A cohort of healthy controls was selected from the institutional echocardiography storage database of our Center. It comprised individuals who underwent transthoracic Doppler echocardiography and laboratory examination between January 2010 and December 2015 at the Echocardiography Lab of our Institution. We included healthy individuals without structural heart disease or cardiovascular risk factors, with normal systolic function (LVEF ≥ 50%), and no signs of structural or electrical cardiac alterations. These individuals were matched for age and sex with the HTx cohort.

Baseline data on demographics, clinical characteristics, medical history, medications, lifestyle habits, and cardiac test results were collected from medical records for both the HT cohort and healthy control cohort. The study was conducted in accordance with the principles of the Declaration of Helsinki and the Declaration of Istanbul. The study was approved by the Ethics Committee for Clinical Trials of the Province of Padua, Italy. Given the retrospective, observational, non‐interventional nature of the study, patients were not asked for specific informed consent.

### 2.2 Echocardiographic Measurements

Transthoracic Doppler echocardiography examination performed during the follow‐up admission at 1 year after HT was retrospectively reviewed from the institutional echocardiography storage database of our Center. Data were collected from medical reports when available. If not available, echocardiographic images were reviewed by two independent echocardiographic readers (M.C. and A.G.) who were blinded to patients’ medical history. Measurements were performed offline by a reader using a vendor‐independent software package (ComPACS, MediMatic Srl, Genoa, Italy) at the time of data collection.

Two‐dimensional and Doppler echocardiographic images were obtained from standard parasternal, apical, and subcostal views. Additionally, pulse‐wave tissue Doppler imaging (TDI) was performed to assess diastolic function [13]. The LV end‐systolic volume (ESV), end‐diastolic volume (EDV), and LVEF were calculated from the conventional apical 2‐ and 4‐chamber images using biplane Simpson's technique.

Pulsed‐wave Doppler parameters included transmitral peak rapid filling and atrial velocity (E and A), E‐wave deceleration time (DT), and E/A ratio. TDI parameters included peak systolic velocity (s’ –index of global systolic function) and early and late diastolic velocities at the septal mitral annulus (e’ and a’, respectively) [13]. Systolic and diastolic blood pressure and heart rate were also recorded. All measurements were performed under stable hemodynamic conditions.

### 2.3 VAC and PV Derived Parameters

**FIGURE 1:**
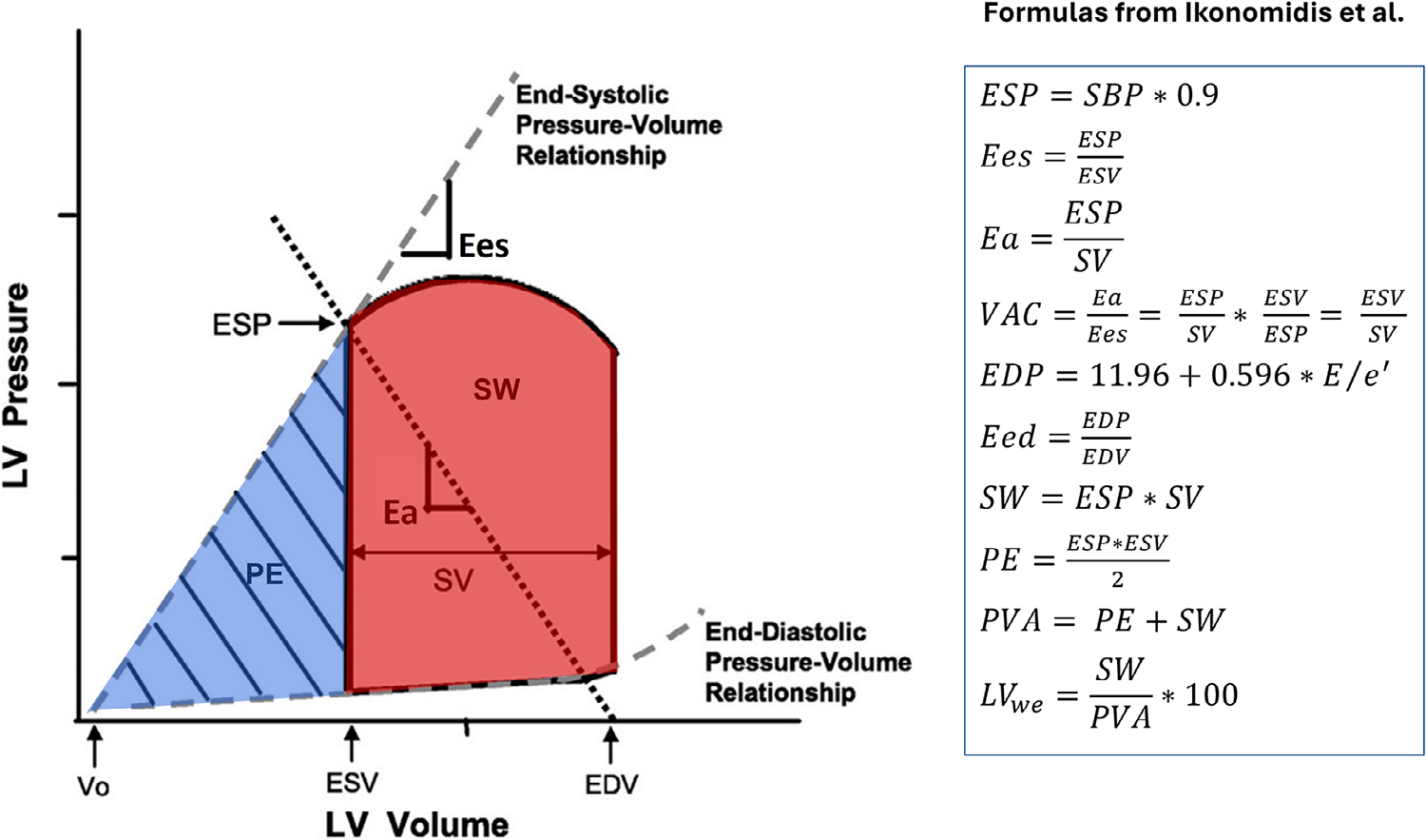
Left ventricle pressure‐volume diagram from which effective arterial elastance (Ea) and end‐systolic elastance (Ees) are derived. Ea is calculated from the ratio between end‐systolic pressure (ESP—approximated as systolic blood pressure [SBP] * 0.9) and stroke volume (SV), and represents the negative slope of the line joining the end‐diastolic volume and the end‐systolic pressure point. Ees is calculated from the ratio between ESP and end‐systolic volume (ESV), and represents the slope of the end‐systolic pressure‐volume relationship passing through the volume intercept with x‐axis (V_0_). Ventricular‐arterial coupling (VAC) is defined as the ratio between Ea and Ees and describes the interaction between the vascular and heart systems. The red area represents cardiac stroke work (SW), which is the useful fraction of ventricular energy delivered to the arterial system for maintaining forward blood flow and providing adequate transport of oxygen and nutrients to peripheral organs. The blue area represents the potential energy (PE), which is the elastic energy stored in the ventricle at the end of the systole and is dissipated as heat during isovolumetric relaxation. Pressure‐volume area (PVA), the sum of stroke work and potential energy, represents the total mechanical energy generated during systole for a given contractility and loading condition. Left ventricle work efficiency (LV_we_) is determined as the percentage of the PVA converted to SW. Under normal conditions, SW generation is maximal when the Ea/Ees ratio equals 1, while maximal cardiac efficiency (LVwe) is achieved when the Ea/Ees ratio equals 0.5. Modified with permission from Chantler et al. [24].

Ventricular and arterial function were measured non‐invasively using simplified formulae applied in daily clinical practice as proposed by Ikonomidis et al. [14]. All variables are derived from the PV loop, and their relationship and meaning are represented in Figure 1.

### 2.4 Outcome and Follow‐Up

Patients were followed up from the date of transplantation until the date of death or cardiac retransplant. The end date of the follow‐up period was December 2023. Survival was considered from 1 year post‐HT. The primary outcome regarded cardiac mortality, defined as a composite of retransplant or death due to graft failure, rejection, acute coronary syndrome/CAV. Patients who experienced a non‐cardiac death were censored. Follow‐up data were obtained by reviewing medical records and conducting interviews during medical visits.

### 2.5 Statistical Analysis

Continuous variables are expressed as mean ± standard deviation if normally distributed or as median with interquartile range (IQR) if skewed, and compared using the Student's *t*‐test or Mann‐Whitney test accordingly; categorical variables are presented as proportions and compared using the Chi‐square test.

First, we compared clinical variables, echocardiographic measurements, and PV‐derived parameters between HT patients and healthy controls.

Second, we stratified HT patients into groups, distinguishing between those whose values for VAC, Ea, and Ees exceeded the median value and those that were lower than the median. Then, a comparative analysis of VAC and its constituent parameters in relation to clinical outcome was performed.

Cumulative patient survival was estimated using the Kaplan‐Meier method, and differences in survival by VAC, Ea, and Ees groups were compared using the log‐rank test. Additionally, other variables were compared according to VAC, Ea, and Ees groups.

Adjusted associations between factors and the clinical endpoint were examined using univariable and multivariable Cox proportional hazard regression models.

Univariable Cox regression analysis was performed for clinical variables, as well as 1‐year post‐transplant echocardiographic and PV variable surrogates. Variables with *p* < 0.05 in univariable analysis were considered for inclusion in the multivariable analysis. Backward stepwise multivariable regression was performed to select the most predictive variables. Hazard ratios (HRs) and 95% confidence intervals (CIs) were calculated. To evaluate the incremental value of Ea and Ees on top of clinical and standard echocardiographic parameters, the overall C‐statistic was calculated as proposed by Harrell et al. [15] as an analogue of the area under the receiver operating characteristic curve for survival analysis. Furthermore, we assessed the impact of adding Ea and Ees to a basic model using continuous net reclassification improvement. The DeLong test was performed to compare the C‐statistic of different models. All tests were two‐sided, and statistical significance was set at *p* < 0.05. Statistical analyses were performed using IBM SPSS Statistics version 28 (Chicago, SPSS, Inc., Chicago, IL).

## 3 Results

### 3.1 Baseline Characteristics

**FIGURE 2:**
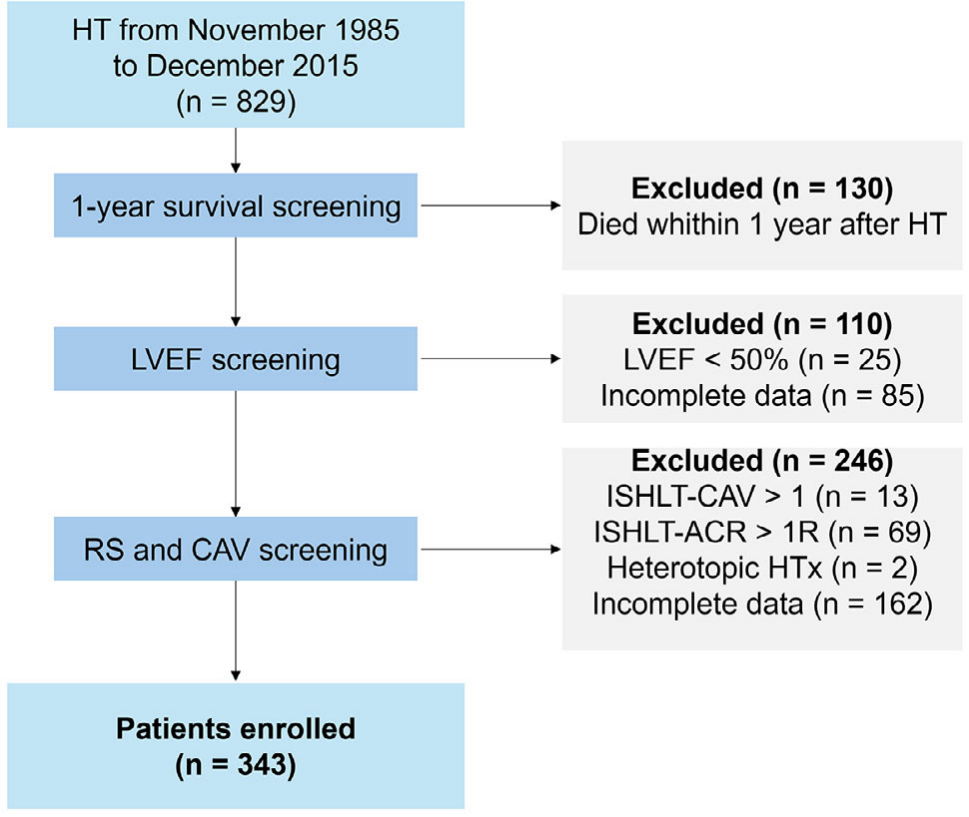
Study flow chart.

From November 1985 to December 2015, a total of 829 HT were performed at our institution. Of these, 486 patients were excluded, and the final HT cohort consisted of 343 patients (Figure 2).

Baseline characteristics of the HT cohort are shown in Table 1. Overall, the cohort was mainly represented by males (75%), and sex mismatch was observed in 118 (34%) patients. The median age of the recipient and donor was 55 (41–62) and 36 (23–48) years, respectively, with a median donor ischemic time of 180 (138–225) min. Of note, combined heart–kidney transplant was performed in 2 (0.5%) patients.

**TABLE 1:** Baseline characteristics of patients who underwent heart transplantation.

Variable	
Sex male *n* (%)	258 (75)
Age at HT, y	55 (41–62)
BSA, m^2^	1.8 (1.6–1.9)
BMI, kg/m^2^	24.2 (20.4–25.0)
***Transplant‐related characteristics***	
Donor age, y	36 (23–48)
Donor BMI, kg/m^2^	24.2 (22.5–26.0)
Donor ischemic time, min	180 (138–225)
Sex mismatch, *n* (%)	118 (34)
Combined heart‐kidney transplant, *n* (%)	8 (2.3)
Ischemic heart disease, *n* (%)	115 (34)
***Laboratory variables***	
Hb, g/dL	12.5 (11.1–13.8)
HCT, %	37.8 (34.0–41.2)
GFR, mL/min	35.5 (24–50)
Azotemia, mg/dL	10.9 (8.1–15.1)
Aspartate aminotransferase, IU/L	23 (19 ‐28)
Alanine aminotransferase, IU/L	19 (14–25)
Na+, mEq/L	140 (138–142)
K+, mEq/L	4.2 (3.8–4.5)
***Medical history***	
Diabetes, *n* (%)	63 (19)
Hypertension, *n* (%)	218 (64)
Hypercholesterolemia, *n* (%)	113 (34)
Obesity, *n* (%)	38 (11)
***Medications***	
Ciclosporine, *n* (%)	327 (97)
Everolimus, *n* (%)	72 (21)
Tacrolimus, *n* (%)	10 (3)
Azatioprine, *n* (%)	89 (26)
Prednisone, *n* (%)	191 (55)
Methylprednisolone, *n* (%)	2 (1)
Mycophenolate mofetil, *n* (%)	124 (36)
ACE‐I/ARB, *n* (%)	101 (34)
CCB, *n* (%)	50 (17)
MRA, *n* (%)	41 (13)
Beta‐blocker, *n* (%)	27 (9)
Diuretics *n* (%)	218 (72)
Statin, *n* (%)	137 (46)

*Note:* Data are presented as *n* (%), mean ± standard deviation, or median (25^th^–75^th^ percentiles).

Abbreviations: ACE‐I, angiotensin‐converting enzyme inhibitor; ARB, angiotensin receptor blockers; BMI, body mass index; BSA, body surface area; CCB, calcium channel blockers; GFR, glomerular filtration rate; Hb, hemoglobin; HCT, hematocrit; HT, Heart transplant; MRA, aldosterone receptor antagonists.

#### 3.1.1 Echocardiographic and PV Derived Parameters in HT and Healthy Controls

EDV, ESV, and SV were lower in HT patients compared to controls, while LV mass and LV thickness were higher. On the other hand, LVEF was similar between the two groups. Regarding diastolic function, in HT patients, E wave, e’, E/A, and E/e’ were higher, and DT was shorter than in controls. Echocardiographic data are shown in Table 2.

**TABLE 2:** Comparison of echocardiographic parameters between healthy controls and HT cohort.

Variable	Healthy controls cohort (*n* = 100)	Heart transplant cohort (*n* = 343)	*p* value
LVEDD, mm	48 (43–50)	25 (23–27)	<0.0001
LVESD, mm	31 (29–34)	14 (13–16)	<0.0001
LVPWT, mm	8 (7–9)	11 (10–12)	<0.0001
LV mass index, g/m^2^	81 (74–86)	95 (79–117)	<0.0001
LV EDV, mL/m^2^	60 (49–75)	48 (41–56)	<0.0001
LV ESV, mL/m^2^	24 (19–30)	18 (14–21)	<0.0001
SV, mL/m^2^	36 (31–47)	31 (26–35)	<0.0001
EF, %	60 (58–66)	63 (59–67)	0.130
Peak E velocity, cm/s	62 (54–68)	79 (56–90)	<0.0001
Peak A velocity, cm/s	56 (52–58)	48 (39–58)	<0.0001
DT, ms	201 (191–220)	165 (140–198)	<0.0001
e’ average, cm/s	7.2 (7.0–8.8)	11 (8.0–13.0)	<0.0001
a’ average, cm/s	8.6 (8.2–9.4)	7 (6.0–8.0)	<0.0001
S’ average, cm/s	7.5 (7.1–8.0)	8.8 (7.8–9.4)	<0.0001
E/A	1.12 (0.9–1.22)	1.63 (1.31–2.20)	<0.0001
e/e’	6.4 (6.0–7.2)	7.4 (5.8–10.4)	<0.0001

*Note:* Data are presented as *n* (%) or median (25th–75th percentiles).

Abbreviations: a’, late diastolic annular tissue velocity; DT, deceleration time; e’, early diastolic mitral annular tissue velocity; E/A, early to late diastolic transmitral flow velocity; E/e’, LV pressure filling; EDV, end‐diastolic volume; EF, ejection fraction; ESV, end‐systolic volume; LVEDD, left ventricular end‐diastolic diameter; LVESD, left ventricular end‐systolic diameter; LVPWT, left ventricular posterior wall thickness; s’, systolic tissue velocity; SV, stroke volume.

Data regarding PV‐derived parameters are shown in Table 3. Ea was higher in HT recipients than in controls (4.03 [IQR: 3.42–4.80] mmHg/mL/m^2^ vs. 2.78 [IQR: 2.24–3.42] mmHg/mL/m^2^, *p* < 0.0001). Ees was also higher (6.75 [IQR: 5.54–8.41] mmHg/mL/m^2^ vs. 4.28 [IQR: 3.34–5.46] mmHg/mL/m^2^, *p* < 0.0001) in HT recipients than controls as well, while VAC was similar (0.66 [IQR: 0.42–0.73] vs. 0.59 [IQR: 0.49–0.71], *p* = 0.110) between the two groups. Regarding cardiac energetics, pressure‐volume area (PVA), stroke work (SW), and potential energy (PE) were lower in HT recipients than in healthy controls (*p* < 0.001), while left ventricular work efficiency (LV_we_) was similar between the two groups (*p* = 0.104).

**TABLE 3:** Comparison of pressure‐volume derived parameters between healthy controls and HT.

Variable	Healthy controls cohort (*n* = 100)	Heart transplant cohort (*n* = 343)	*p* value
Ea, mmHg/mL/m^2^	2.78 (2.24–3.32)	4.03 (3.42–4.80)	<0.0001
Ees, mmHg/mL/m^2^	4.28 (3.34–5.21)	6.75 (5.54–8.41)	<0.0001
VAC	0.66 (0.42–0.73)	0.59 (0.48–0.70)	0.105
SW, mmHg*mL/m^2^	4015 (3324–4974)	3651 (3023–4445)	0.017
PE, mmHg*mL/m^2^	1203 (907–1544)	1079 (838–1338)	0.020
PVA, mmHg*mL/m^2^	5063 (4336–6382)	4717 (4020–5680)	0.015
LV_we_, %	75 (73–82)	77 (74–80)	0.104
Eed, mmHg/mL/m^2^	0.15 (0.14–0.19)	0.35 (0.30–0.43)	<0.0001
ESP, mmHg	108 (99–105)	117 (108–135)	<0.0001

*Note:* Data are presented as *n* (%) or median (25th–75th percentiles).

Abbreviations: Ea, arterial elastance; Eed, left ventricle end diastolic elastance; Ees, left ventricle end systolic elastance; ESP, end‐systolic pressure; LVwe, left ventricle work efficiency; PE, potential energy; PVA, pressure‐volume area; SW, stroke work; VAC, ventricle arterial coupling.

### 3.2 Association of VAC and Its Components With Cardiac Mortality

**FIGURE 3:**
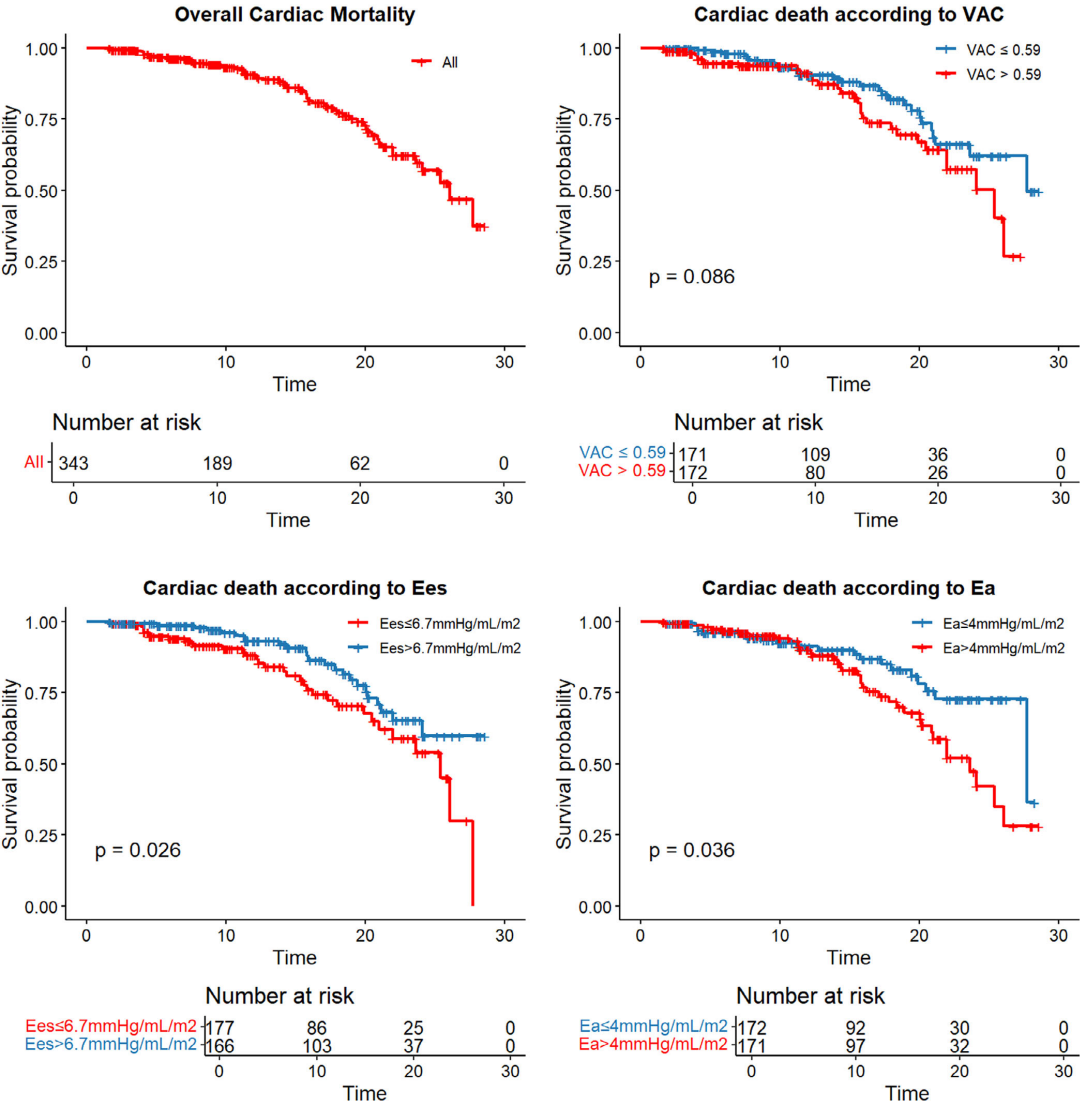
Kaplan‐Meier curves for 30‐year cumulative graft and patient survival according to ventricular‐arterial coupling (VAC), ventricular elastance (Ees), and arterial elastance (Ea) value. (A) entire population; (B) according to VAC above or below 0.59; (C) according to Ees above or below 6.75 mmHg/mL/m^2^; (D) according to Ea above or below 4.0 mmHg/mL/m^2^.

After a median follow‐up of 11.3 years (IQR: 6.29–17.75 years), the cardiac mortality endpoint, conditional on surviving the first year, was observed in 58 patients (17%), with an overall median survival of 26.1 years (Figure 3A).

One‐year conditional survival tended to be lower in patients with higher VAC values but not significantly (median survival 27 vs. 25 years, Log rank *p* = 0.086) (Figure 3B). Furthermore, survival conditional on surviving the first year was lower in HT recipients with Ees ≤ 6.75 mmHg/mL/m^2^ (median survival 21 vs. 24 years, Log rank *p* = 0.026) (Figure 3C) and in patients with Ea >4.0 mmHg/mL/m^2^ (median survival 23 vs. 27 years, Log rank *p* = 0.036) (Figure 3D).

In the univariable Cox regression analysis, VAC was not associated with worse outcome (*p* = 0.088), while Ea >4 mmHg/mL/m^2^ and Ees ≤ 6.7 mmHg/mL/m^2^ were significantly associated with cardiac mortality (*p* = 0.038 and *p* = 0.028, respectively). Other echocardiographic variables (LV‐ESV, EDV, EF, SV) were not associated with outcome (Table S1).

**FIGURE 4:**
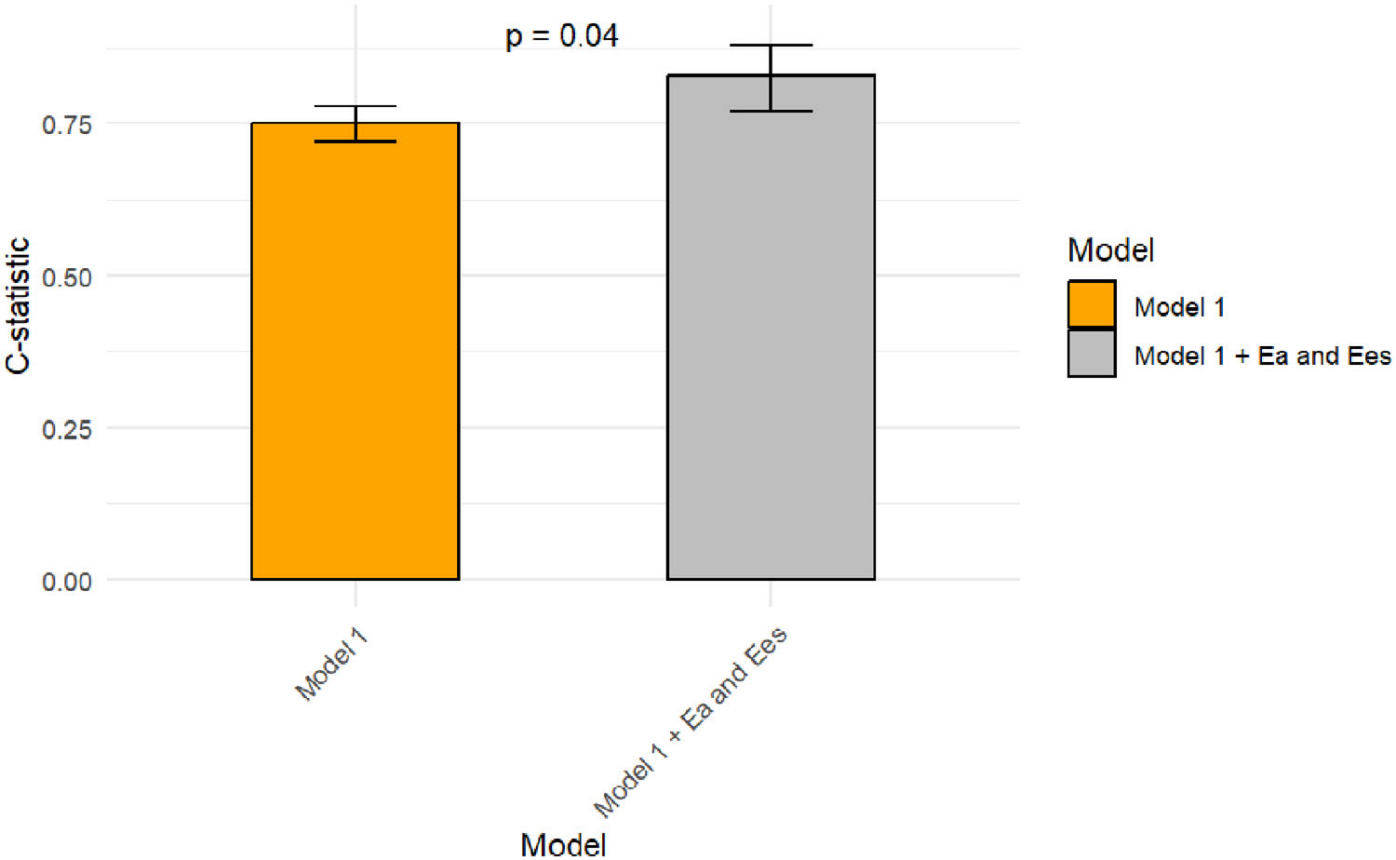
Performance of the two survival prediction models among HT patients. Age at transplant, donor ischemic time, L‐VAD before transplant, and hemoglobin level were identified as independent predictors of cardiac mortality (referred to as Model 1) (χ [2 **]** = 55.374, *p* < 0.001). The inclusion of Ea and Ees into Model 1 resulted in an increased prognostic value (χ [2 **]** = 73.243, *p* < 0.001, De Long *p* = 0.04), improving the prediction of survival among HT patients.

After including clinical variables in a multivariable hazard model, recipient and donor age at transplant, donor ischemic time, L‐VAD before transplant, and recipient diabetes status were associated with cardiac mortality, and the model showed a discrete predictive accuracy (χ^2^ = 55.374, *p* < 0.001). After adding Ea and Ees to the multivariable model, both Ea and Ees were independently associated with worse outcome (HR = 3.07 [95% CI 1.46–6.47], *p* 0.003; HR = 2.35 [95% CI 1.11–4.97], *p* = 0.025, respectively). The second model showed a better predictive accuracy (χ^2^ = 73.243, *p* < 0.001) compared to the model with only clinical variables (DeLong *p* = 0.041). This indicates that including Ea and Ees improves the prediction of survival among HT patients (Figure 4).

When comparing HT recipients according to their Ees values, Ea and Eed were higher in patients with Ees>6.75 mmHg/mL/m^2^, while VAC, SW, PE, PVA, and L_we_ were lower. Moreover, EDV, ESV, and SV were lower in patients with Ees >6.75 mmHg/mL/m^2^, while LVEF was higher (Table S2).

When comparing HT recipients according to their Ea values, Eed and PVA were higher in patients with Ea> 4.0 mmHg/mL/m^2^, while SW, PE, PVA, LV EDV, LV ESV, and SV were lower. VAC was similar between groups (Table S3).

Other information regarding patients’ characteristics according to VAC is reported in Table S4.

## 4 Discussion

The present study confirmed in a large cohort that HT patients surviving their first year after transplantation exhibit increased Ea and Ees compared to healthy controls. Moreover, for the first time, we demonstrated the prognostic role of Ea and Ees in HT patients with normal LVEF. Compared to previous studies, our research has several distinctive features. Firstly, our cohort was significantly larger, making the findings more robust. Secondly, we used a non‐invasive method to assess Ea and Ees. Although less precise than invasive techniques, this method allows for easy evaluation of ventricular‐vascular interactions and facilitates repeated measurements over time. Thirdly, we evaluated the association between different profiles of Ea and Ees and cardiac mortality over an extended period of follow‐up. Fourthly, the HT cohort in our study was carefully selected to eliminate confounding factors that could reduce the accuracy of the analysis. In fact, patients were included if they survived at least 1 year after HT. It is well known that the first year after HT is critical due to the high risk of death from primary graft failure, rejection, and infection [16, 17, 18]. Additionally, patients were included only if they had mild signs of CAV or rejection and an LVEF above 50%. This selection criterion explains why the median survival, conditional on surviving the first year, was 26 years in our cohort, compared to the 14.8 months reported by international registries [19]. In fact, it is known that 22.7% of HT patients require rejection treatment in the first year, and they have a higher risk of mortality in subsequent years [20]. Similarly, it is estimated that CAV is present in approximately 8% of HT patients within the first year post‐transplantation, significantly impacting long‐term survival [21, 22].

Our results have some potential clinical implications. The possibility of identifying alteration of LV physiology at an early stage, when LV‐EF is still normal, and with a non‐invasive method, may justify further investigations or a prompt adjustment of medical therapy to prevent complications at an irreversible stage. In particular, an increase in Ea can be interpreted as a sign of inadequate management of arterial hypertension and may warrant an up‐titration of medical therapy. On the other hand, a reduction of Ees may indicate the presence of an underlying subtle complication requiring further tests.

### 4.1 Increased Values of Ea and Ees Compared to Healthy Controls

Given that there is no established normal value of VAC [23], the HT cohort was first compared to a cohort of healthy subjects to identify analogies or differences between the two groups [14]. The conclusion was that VAC did not differ between the two groups, but Ea and Ees were higher in the HT cohort.

In the control group, the median values of Ea and Ees were similar to those reported in previous studies involving healthy subjects using the same non‐invasive method employed in our study [8, 24–26]. Regarding HT patients, only three previous studies evaluated Ea, Ees, and VAC in this population, and they were heterogeneous in their measurements [7, 8, 10]. Invasive measurements were used by Arnoult et al. [7], reporting median values of indexed Ees, Ea, and VAC of 2.58, 2.53 mmHg/mL/m^2^ and 0.96, respectively. A non‐invasive method was used by Milani et al. [8] and Mehra et al. [9], employing a single‐beat method as validated by Chen et al. [27]. In both studies, Ea was calculated as end‐systolic pressure (ESP)/SV, while Ees was derived using stroke volume (SV), LVEF, and timing of ejection phases. The former study (58 patients) reported mean Ea, Ees, and VAC values of 2.96, 3.12 mmHg/mL, and 0.95, respectively. The latter study (40 patients) reported mean values of Ea, Ees, and VAC of 2.58, 2.51 mmHg/mL, and 0.96, respectively. In our study, the median value of Ea was slightly higher, and we can affirm that our value can be considered more accurate than those of the other studies for three reasons: the Ea calculation method matched the previous studies; the control group's median Ea aligned with values from other healthy cohorts; and the study had a larger patient population (343 patients) with uniform echocardiographic evaluation conducted 1 year after heart transplantation.

Regarding Ees, in our study, the median value was higher than in the previous report. This discrepancy is likely due to the use of a simplified formula (Ees = ESP/[LV‐ESV]), which, as noted by Chen et al. [27], tends to overestimate Ees, especially at higher values, and less correlates with invasive measurements compared to the single‐beat method. Despite its lower accuracy, the simplified formula remains a practical tool in clinical settings, though further studies are needed to clarify its role in managing HT patients.

The observed increase in Ea and Ees in the HT cohort aligns with prior findings, reflecting the altered physiology of the transplanted heart [8, 9]. Elevated Ea may result from increased afterload—due to aortic stiffness, immunosuppressive therapy, chronic kidney disease, or denervation—or from reduced SV [28]. Regarding the decrease in SV, our study population exhibited normal cardiac function (LVEF>50%), and the reduction may be attributed to the concentric remodeling occurring after HT, typically consequent to elevated arterial blood pressure and immunosuppressive agents [29, 30, 31]. In particular, elevated blood pressure leads to an increase in afterload, stimulating hypertrophy similarly to healthy patients, through the conversion of mechanical stimuli into biochemical events, resulting in the release of growth factors and angiogenesis [32, 33, 34]. Immunosuppressive agents, in particular calcineurin inhibitors, activate different biological pathways that lead to cardiomyocyte hypertrophy [35].

In our study, LV mass and thickness were higher in HT recipients than in controls, while LV volumes were lower, confirming the aforementioned process. The concentric remodeling may further explain the reduction of cardiac energetics parameters SW, PE, and PVA, but not LV_we_. In fact, in physiological circumstances, the ventricle operates toward a metabolic optimization criterion by enhancing ventricular efficiency. Similarly, in the transplanted heart, the hypertrophic status leads to a reduction of ESV and SV, resulting in a lower PE and SW. On the other hand, Ees is an index of myocardial contractility, reflecting the ability of the left ventricle (LV) to eject blood against a given pressure. A decrease in Ees is generally associated with reduced myocardial contractility, and such an alteration can be found even within normal LVEF [14, 36].

### 4.2 Association Between Increased Ea and Reduced Ees With Cardiac Mortality

In the aforementioned study, Latus et al. [10] enhanced an impaired VAC, Ea, and Ees both at rest and under inotropic stimulation in HT patients, concluding that the prognostic relevance of such impairment remained unknown. In our study, we demonstrated that an increased Ea and a reduced Ees were associated with worse outcomes. The association between increased afterload and cardiac death can be explained similarly to the natural history of hypertensive heart disease (HHD) [37, 38, 39, 40]. In fact, in HHD, the exposure to increased afterload leads to concentric remodeling and, after 5–10 years, progresses to HF and cardiac death [40]. Similarly, in our cohort, the increase in cardiac mortality among patients with higher Ea becomes evident after 10 years of follow‐up, as shown in Figure 3. These findings have two main clinical implications. First, high‐risk patients can be detected early, even among those with normal or mildly elevated blood pressure. In fact, even when blood pressure appears to be well‐controlled, a hypertensive patient with concentric remodeling due to various causes tends to have lower SV and, consequently, higher Ea. Second, many drugs have been shown to effectively improve arterial function [41, 42, 43], suggesting that an early management strategy can be initiated in high‐risk patients to improve long‐term outcomes.

The association between reduced Ees and cardiac death is consistent with findings in other cardiac conditions, where low Ees indicates decreased contractility and poor prognosis [14]. However, the cause of reduced Ees is unclear. On one hand, it may reflect an increasing afterload sensitivity resulting in afterload mismatch [44, 45]. On the other hand, a reduction in Ees can be an early sign of major complications of HT, particularly CAV. Although coronary angiography is standard for CAV surveillance, it may miss early atherosclerosis [21]. In this regard, Tuzcu et al. [22] demonstrated that intravascular ultrasound detected lesions in 55% of patients missed by angiography within the first month post‐transplant, with 47% developing new lesions within a year, correlating with a higher risk of death or myocardial infarction.

Currently, no echocardiographic parameters reliably detect early CAV, and reduced LV‐EF is typically seen only in advanced cases [46]. A decline in Ees may serve as an early marker, indicating impaired contractility from microvascular dysfunction or progressive atherosclerosis. Identifying early signs like reduced Ees could help detect subclinical CAV and guide more intensive therapies. Although our study did not explore this, future research should investigate this potential.

### 4.3 Limitation

Our study has limitations that deserve to be mentioned. Firstly, it was a single‐center retrospective study, and these findings need to be confirmed in multicentric registries to enhance generalizability.

Secondly, deriving hemodynamic parameters using echocardiography presents certain challenges, including issues related to body habitus, operator dependence, and, given the extended duration of our study, the accuracy of software and hardware used for measurements. Moreover, a comparison between invasive and non‐invasive methods for measuring PV variable surrogates has not been tested in HT patients. Although the use of a non‐invasive method to measure and calculate VAC components and cardiac energetic parameters is beneficial for clinical practice, it may limit measurement accuracy [47, 48]. In fact, the simplification of Chen's formula used to calculate VAC makes it a derivative of LV ejection fraction [14, 24, 49]. This may explain why VAC was not able to stratify outcomes, even if it was theoretically expected. Furthermore, Ea and Ees calculated non‐invasively may be more load‐dependent, limiting their clinical impact.

Thirdly, the association between Ea and Ees with cardiac mortality was demonstrated over a 30‐year follow‐up period. However, the strength of a single measurement over such an extended timeframe is limited. Therefore, the association identified in this study does not imply clear causation between echocardiographic parameters and cardiac outcomes. Nevertheless, the potential to identify an early marker of graft dysfunction that is easily detectable in clinical practice justifies further research in this field. Additional studies are needed to evaluate how ventricular‐vascular interactions change over time after HT, how these changes impact outcomes, and whether strategies aimed at improving Ea and Ees can improve survival. In conclusion, we highlighted the impact of VAC and its components on cardiac mortality in HT patients with normal LVEF. A higher value of Ea, reflecting an increased arterial stiffness, and a lower value of Ees, reflecting a decreased myocardial contractility, were associated with a poorer prognosis. On the contrary, VAC did not correlate with long‐term outcome in HT patients.

## Conflicts of Interest

The authors declare no conflicts of interest.

## Supporting information



Supporting Information

## Data Availability

Data are available upon reasonable request.
