# Supplementary material for: Impact of Left Ventricular‐Vascular Interaction on Long‐Term Outcome After Heart Transplantation
Source: Clin Transplant. 2025 May 13;39(5):e70178. doi: 10.1111/ctr.70178 (PMC12072248; doi:10.1111/ctr.70178)
Supplement: Supplementary file 1 — Supporting Information [file CTR-39-e70178-s001.docx]

# **Supplementary appendix**

## **Methods**

### **Coronary angiography**

All HT recipients underwent a coronary angiography (CA) as a part of routine assessment to evaluate the onset and development of CAV. More specifically, CA is performed close after surgery, annually at 1, 2 and 3 years after HT, then biannually. Each angiographic report included a description of the maximum stenosis at the level of the left main artery, primary vessels, and secondary branch vessels. CAV was defined and classified according to the standard criteria of International Society for Heart and Lung Transplantation (ISHLT).^14^ Given that the classification has changed over time, angiographic images were reviewed by two independent readers (N.P., C.T.), who were blinded to the patients’ medical history, and reclassified according to the latest 2010 ISHLT classification. Patients with a ISHLT CAV of grade 0 (no detectable angiographic lesion) or 1 (angiographic left main <50%, or primary vessel with maximum lesion of 70% or any branch stenosis <70% without allograft dysfunction) at 1 year from HT were included in the cohort.

### **Endomyocardial biopsy and rejection**

Monitoring of acute rejection was performed by endomyocardial biopsy (EMB) according to the established protocol of our Institution. After HT, patients underwent EMB once a week in the first month, once every two weeks until the third month, and once a month until the first year; in the presence of a grade 2R rejection, the biopsy was performed again after 10-15 days; no EMB was performed after the first year, except for the presence of clinical suspicion of rejection. Graft rejection was classified according to the ISHLT- Acute Cellular Rejection (ACR) grading.^15^ Given that the classification has changed over time, endomyocardial biopsies were reviewed by two independent readers (A.A, M.F.), who were blinded to the patients’ medical history, and reclassified according to the latest 2021 ISHLT classification. Patients with a ISHLT ACR of grade 0 (no rejection) or 1R (mild rejection, interstitial and/or perivascular infiltrate with up to one focus of myocyte damage) at 1 year from HT were included in the cohort.

# **Supplementary tables**

## **Supplementary table 1**

| **Table S1. Univariable and multivariable Cox Regression for all-cause mortality after heart transplantation** | | | | | | |
| --- | --- | --- | --- | --- | --- | --- |
|  | **Univariate** | | | **Multivariate** | | |
|  | **HR** | **95% CI** | **p value** | **HR** | **95% CI** | **p value** |
| VAC >0.59 | 1.58 | 0.93 – 2.66 | 0.088 |  |  |  |
| Ees ≤ 6.7mmHg/mL/m^2^ | 1.82 | 1.08 – 3.07 | ***0.028*** | 3.07 | 1.46 – 6.47 | ***0.003*** |
| Ea >4mmHg/mL/m^2^ | 1.80 | 1.06 – 3.09 | ***0.038*** | 2.35 | 1.11 – 4.97 | ***0.025*** |
| Eed | 10.8 | 0.54 – 15.31 | 0.651 |  |  |  |
| SW | 1.09 | 0.83 – 1.67 | 0.476 |  |  |  |
| PE | 1.50 | 0.85 – 2.54 | 0.583 |  |  |  |
| PVA | 1.08 | 0.72 – 1.48 | 0.700 |  |  |  |
| LV_we_, (%) | 1.00 | 0.94 – 1.06 | 0.387 |  |  |  |
| LVEF | 0.97 | 0.95 – 1.01 | 0.089 |  |  |  |
| LV EDV | 0.99 | 0.97 – 1.03 | 0.499 |  |  |  |
| LV ESV | 1.01 | 0.98 – 1.04 | 0.700 |  |  |  |
| SV | 0.97 | 0.93 – 1.01 | 0.120 |  |  |  |
| ESP | 1.01 | 0.99 – 1.03 | 0.501 |  |  |  |
| Age at transplant | 1.06 | 1.03 – 1.08 | ***<0.001*** | 0.99 | 1.00 – 1.07 | 0.074 |
| Donor age | 1.06 | 1.04 – 1.08 | ***<0.001*** | 1.05 | 1.02 – 1-08 | ***<0.001*** |
| Recipient male sex | 2.06 | 1.01 – 4.20 | ***0.047*** | 0.91 | 0.36 – 2.31 | 0.839 |
| Donor male sex | 0.76 | 0.45 – 1.28 | 0.300 |  |  |  |
| Sex mismatch | 1.56 | 0.92 – 2.64 | 0.101 |  |  |  |
| BSA | 2.62 | 0.69 – 9.89 | 0.222 |  |  |  |
| BMI | 1.03 | 0.96 – 1.11 | 0.436 |  |  |  |
| Donor BMI | 1.07 | 0.96 – 1.19 | 0.287 |  |  |  |
| Donor ischemic time | 1.01 | 1.00 – 1.01 | ***0.002*** | 1.01 | 0.99 – 1.02 | 0.079 |
| L-VAD | 6.85 | 2.66 – 17.70 | ***<0.001*** | 1.76 | 0.41 – 7.52 | 0.445 |
| Ischemic heart disease | 2.47 | 1.45 – 4.21 | ***<0.001*** | 1.40 | 0.65 – 3.03 | 0.389 |
| Combined heart-kidney | 0.68 | 0.16 – 2.79 | 0.603 |  |  |  |
| Tacrolimus | 2.92 | 0.90 – 9.48 | 0.074 |  |  |  |
| Everolimus | 1.70 | 0.98 – 2.84 | 0.059 |  |  |  |
| Ciclosporin | 0.25 | 0.09 – 0.71 | ***0.009*** | 0.15 | 0.03 – 0.64 | 0.010 |
| Azatioprin | 0.78 | 0.41 – 1.48 | 0.453 |  |  |  |
| Prednisone | 3.32 | 1.85 – 5.97 | ***<0.001*** | 3.87 | 1.81 – 8.29 | <0.001 |
| Methilprednisolone | 1.19 | 0.16 – 8.72 | 0.890 |  |  |  |
| Mycophenolate mofetil | 1.46 | 0.85 – 2.49 | 0.204 |  |  |  |
| ACE-I/ARB | 0.58 | 0.32 – 1.06 | 0.079 |  |  |  |
| CCB | 0.85 | 0.41 – 1.76 | 0.702 |  |  |  |
| MRA | 0.94 | 0.29 – 3.04 | 0.962 |  |  |  |
| Diuretic | 3.87 | 1.78 – 8.40 | ***<0.001*** | 2.55 | 1.01 – 5.95 | 0.029 |
| Beta-blocker | 1.03 | 0.46 – 2.31 | 0.964 |  |  |  |
| Statin | 1.06 | 0.60 – 1.88 | 0.824 |  |  |  |
| Diabetes | 1.20 | 0.60 – 2.37 | 0.621 | 0.87 | 0.39 – 1.91 | 0.726 |
| Hypertension | 1.02 | 0.59 – 1.76 | 0.984 |  |  |  |
| Hypercholesterolemia | 1.02 | 0.58 – 1.78 | 0.990 |  |  |  |
| Obesity | 1.17 | 0.55 – 2.48 | 0.720 |  |  |  |
| GFR | 0.99 | 0.98 – 1.01 | 0.478 |  |  |  |
| Deceleration time | 1.00 | 0.99 – 1.01 | 0.134 |  |  |  |
| E/A | 0.56 | 0.24 – 1.34 | 0.195 |  |  |  |
| E/e’ | 1.09 | 0.86 – 1.39 | 0.450 |  |  |  |
| Mitral regurgitation | 0.74 | 0.42 – 1.31 | 0.300 |  |  |  |
| Tricuspid regurgitation | 0.61 | 0.36 – 1.05 | 0.076 |  |  |  |
| TAPSE | 0.40 | 0.01 – 0.69 | ***0.027*** | - | - | - |
| PAPs | 0.98 | 0.93 – 1.03 | 0.599 |  |  |  |
| Pericardial effusion | 3.94 | 1.88 – 8.28 | ***<0.001*** | - | - | - |
| LVEDD | 0.50 | 0.20 – 1.31 | 0.120 |  |  |  |
| LVPWT | 6.12 | 0.87 – 43.11 | 0.069 |  |  |  |
| Hb | 0.87 | 0.76 – 0.99 | ***0.033*** | - | - | ***-*** |
| Hct | 0.95 | 0.91 – 1.00 | 0.045 |  |  |  |
| Azotaemia | 1.02 | 0.98 – 1.06 | 0.300 |  |  |  |
| AST | 0.99 | 0.97 – 1.01 | 0.488 |  |  |  |
| ALT | 1.01 | 0.98 – 1.02 | 0.598 |  |  |  |
| Na+ | 1.01 | 0.93 – 1.10 | 0.855 |  |  |  |
| K+ | 0.43 | 0.25 -0.74 | ***0.002*** | - | - | - |
| ACE-I: angiotensin-converting enzyme inhibitor; ARB: angiotensin receptor blockers; BSA: body surface index; BMI: body mass index; CCB: calcium channel blockers; Ea: arterial elastance; EDV: end-diastolic volume; Eed: left ventricle end-diastolic elastance; Ees left ventricle end systolic elastance; EF: ejection fraction; ESV: end-systolic volume; GFR= glomerular filtration rate; Hb: hemoglobin; Hct: hematocrit L-VAD: left ventricular assistant device; LVEDD: left ventricle end diastolic diameter; LVPWT: left ventricle posterior wall thickness; LV_we_: left ventricle work efficiency; MRA: aldosterone receptor antagonists; PAPs: pulmonary artery systolic pressure; PVA: pressure-volume area; SV: stroke volume; SW: stroke work; TAPSE: tricuspid annular plane systolic excursion; VAC: ventricular-arterial coupling. | | | | | | |

## **Supplementary table 2**

| **Supplementary Table S2. Ventricular and arterial function according to Ees value** | | | |
| --- | --- | --- | --- |
|  | **Ees ≤ 6.75mmHg/mL** | **Ees > 6.75mmHg/mL** | **p value** |
| VAC | 0.68 (0.58 – 0.76) | 0.50 (0.44 – 0.60) | <0.001 |
| Ea, mmHg/mL/m^2^ | 3.57 (3.13, 4.09) | 4.54 (3.86 - 5.24) | <0.001 |
| Eed, mmHg/mL/m^2^ | 0.33 (0.27 – 0.38) | 0.40 (0.34 – 0.45) | <0.001 |
| SW, mmHg*mL/m^2^ | 3535 (3004 - 4158) | 3388 (2772 - 4062) | 0.100 |
| PE, mmHg*mL/m^2^ | 1281 (1081 - 1523) | 884 (705 - 1074) | <0.001 |
| PVA, mmHg*mL/m^2^ | 5219 (4263 - 6031) | 4324 (3678 - 5398) | 0.004 |
| LV_we_, % | 74.7 (72.5 - 77.4) | 80.0 (76.9 - 81.8) | <0.001 |
| LV EF, % | 59.6 (56.9 - 63.2) | 66.7 (62.5 - 69.2) | <0.001 |
| LV EDV, mL/m^2^ | 56.0 (49.2 – 61.1) | 43.5 (38.3 – 48.8) | <0.001 |
| LV ESV, mL/m^2^ | 21.3 (19.0 - 25.0) | 14.3 (12.0, 16.7) | <0.001 |
| SV, mL/m^2^ | 33.9 (29.6 - 37.0) | 27.8 (24.9 – 32.7) | <0.001 |
| LV EDD, mm | 25 (23 - 27) | 24 (22 - 26) | 0.005 |
| LV PWT, mm | 11 (10 - 12) | 11 (10 - 12) | 0.800 |
| LV IVS, mm | 11 (10 – 13) | 12 (10 - 13) | 0.766 |
| LV mass, mm^3^ | 79 (68 – 91) | 75 (61 – 89) | 0.157 |
| Data are presented as median (25th–75th percentiles). Ea: arterial elastance; EDD: end-end diastolic diameter; EDV: end diastolic volume; Eed: left ventricle end diastolic elastance; Ees: left ventricle end systolic elastance; EF: ejection fraction; ESV: end systolic volume indexed; IVS: interventricular septum; LV: left ventricle; LV_we_: left ventricle work efficiency; PE: potential energy; PWT: posterior wall thickness; PVA: pressure-volume area; SV: stroke volume indexed; SW: stroke work; VAC: Ventricular arterial coupling. | | | |

## **Supplementary table 3**

| **Supplementary Table 3. Ventricular and arterial function according to Ea value** | | | |
| --- | --- | --- | --- |
|  | **Ea ≤ 4.0mmHg/mL** | **Ea > 4.0 mmHg/mL** | **p value** |
| VAC | 0.63 (0.55 – 0.70) | 0.62 (0.51 – 0.72) | 0.213 |
| Ees, mmHg/mL/m^2^ | 5.87 (4.84 – 7.04) | 7.79 (6.50 – 9.71) | <0.001 |
| Eed, mmHg/mL/m^2^ | 0.33 (0.27, 0.39) | 0.40 (0.33, 0.47) | <0.001 |
| SW, mmHg*mL/m^2^ | 4055 (3553 - 4784) | 3226 (2693 - 3861) | <0.001 |
| PE, mmHg*mL/m^2^ | 1170 (902 - 1464) | 993 (808 - 1189) | 0.001 |
| PVA, mmHg*mL/m^2^ | 5309 (4527 - 6060) | 4209 (3628 - 5088) | <0.001 |
| LV_we_, % | 77.8 (74.7 - 81.0) | 76.4 (73.7 - 79.5) | 0.051 |
| LV EF, % | 63.6 (59.6 - 68.0) | 61.8 (58.3 - 66.0) | 0.010 |
| LV EDV, mL/m^2^ | 55 (50 - 61) | 43 (38 - 48) | <0.001 |
| LV ESV, mL/m^2^ | 20.0 (16.0 - 24.0) | 16.0 (13.0 - 19.0) | <0.001 |
| SV, mL/m^2^ | 34.7 (31.7 - 38.0) | 26.01 (22.99 – 29.08) | <0.001 |
| LV EDD, mm | 25 (24 - 28) | 24 (22 - 26) | <0.001 |
| LV PWT, mm | 11 (10 – 12) | 11 (10 - 12) | 0.300 |
| LV IVS, mm | 11 (10 – 12) | 12 (10 – 13) | 0.202 |
| LV mass, mm^3^ | 80 (69 – 90) | 75 (61 – 90) | 0.044 |
| Data are presented as median (25th–75th percentiles). Ea: arterial elastance; EDD: end-end diastolic diameter; EDV: end diastolic volume; Eed: left ventricle end diastolic elastance; Ees: left ventricle end systolic elastance; EF: ejection fraction; ESV: end systolic volume indexed; IVS: interventricular septum; LV: left ventricle; PE: potential energy; PWT: posterior wall thickness; PVA: pressure-volume area; SV: stroke volume indexed; SW: stroke work; VAC: Ventricular arterial coupling. | | | |

## **Supplementary table 4**

| **Supplementary Table 4. Ventricular and arterial function according to VAC value** | | | |
| --- | --- | --- | --- |
|  | **VAC ≤ 0.59** | **VAC > 0.59** | **p value** |
| Ea, mmHg/mL/m^2^ | 3.85 (3.30 - 4.62) | 4.10 (3.55 - 4.85) | 0.095 |
| Ees, mmHg/mL/m^2^ | 8.04 (6.69 - 9.82) | 3.85 (3.30 - 4.62) | <0.001 |
| Eed, mmHg/mL/m^2^ | 0.38 (0.31 - 0.43) | 0.34 (0.28 - 0.45) | 0.298 |
| SW, mmHg*mL/m^2^ | 3783 (3207 - 4633) | 3531 (2875 - 4193) | 0.044 |
| PE, mmHg*mL/m^2^ | 905 (706 - 1120) | 1245 (1047 - 1520) | 0.001 |
| PVA, mmHg*mL/m^2^ | 4709 (4011 - 5707) | 4755 (4020 - 5674) | 0.890 |
| LV_we_, % | 80.5 (78.7 - 82.2) | 74.0 (72.4 - 75.5) | <0.001 |
| LV EF, % | 67.3 (64.9 - 69.8) | 58.7 (56.8 - 60.7) | <0.001 |
| LV EDV, mL/m^2^ | 46.8 (41.0 – 53.1) | 50 .1 (42.3 – 58.9) | 0.002 |
| LV ESV, mL/m^2^ | 15.0 (12.5 - 18.0) | 20.9 (18.0 - 24.0) | <0.001 |
| SV, mL/m^2^ | 32.3 (27.1 – 37.0) | 29.0 (24.8-34.1) | <0.001 |
| LV EDD, mm | 25 (23 – 27) | 24 (23 – 27) | 0.231 |
| LV PWT, mm | 11 (10 – 12) | 11 (10 – 12) | 0.936 |
| LV IVS, mm | 12 (10 – 12) | 11 (10 – 13) | 0.958 |
| LV mass, mm^3^ | 80 (67 – 92) | 76 (63 – 89) | 0.391 |
| Data are presented as median (25th–75th percentiles). Ea: arterial elastance; EDD: end-end diastolic diameter; EDV: end diastolic volume; Eed: left ventricle end diastolic elastance; Ees: left ventricle end systolic elastance; EF: ejection fraction; ESV: end systolic volume indexed; IVS: interventricular septum; LV: left ventricle; LV_we_: left ventricle efficiency; PE: potential energy; PWT: posterior wall thickness; PVA: pressure-volume area; SV: stroke volume indexed; SW: stroke work; VAC: Ventricular arterial coupling. | | | |
